# In Vivo Dynamic Movement of Polymerized Amyloid β in the Perivascular Space of the Cerebral Cortex in Mice

**DOI:** 10.3390/ijms23126422

**Published:** 2022-06-08

**Authors:** Itsuki Hasegawa, Yoko Hirayoshi, Shinobu Minatani, Toshikazu Mino, Akitoshi Takeda, Yoshiaki Itoh

**Affiliations:** Department of Neurology, Osaka City University Graduate School of Medicine, Asahicho 1-4-3, Abenoku, Osaka 545-8585, Japan; c21293u@omu.ac.jp (I.H.); yoko.hirayoshi@omu.ac.jp (Y.H.); s.minatani@omu.ac.jp (S.M.); b21488z@omu.ac.jp (T.M.); akitoshi.takeda@omu.ac.jp (A.T.)

**Keywords:** Alzheimer’s disease, capillary, cerebral amyloid angiopathy, cerebral cortex, penetrating vessel

## Abstract

Disposition of amyloid β (Aβ) into the perivascular space of the cerebral cortex has been recently suggested as a major source of its clearance, and its disturbance may be involved in the pathogenesis of cerebral amyloid angiopathy and Alzheimer’s disease. Here, we explored the in vivo dynamics of Aβ in the perivascular space of anesthetized mice. Live images were obtained with two-photon microscopy through a closed cranial window. Either fluorescent-dye-labeled Aβ oligomers prepared freshly or Aβ fibrils after 6 days of incubation at 37 °C were placed over the cerebral cortex. Accumulation of Aβ was observed in the localized perivascular space of the penetrating arteries and veins. Transportation of the accumulated Aβ along the vessels was slow and associated with changes in shape. Aβ oligomers were transported smoothly and separately, whereas Aβ fibrils formed a mass and moved slowly. Parenchymal accumulation of Aβ oligomers, as well as Aβ fibrils along capillaries, increased gradually. In conclusion, we confirmed Aβ transportation between the cortical surface and the deeper parenchyma through the perivascular space that may be affected by the peptide polymerization. Facilitation of Aβ excretion through the system can be a key target in treating Alzheimer’s disease.

## 1. Introduction

Amyloid β (Aβ), a 40/42 amino acid peptide, is a major component of senile plaque, which is one of the pathological features of Alzheimer’s disease (AD) [1]. Abnormal accumulation of Aβ in the cerebral cortex is regarded as an initial event followed by tau accumulation in the development of AD [1,2,3]. The possible pathways of Aβ efflux include transcytosis through the blood–brain barrier, discharge into the perivascular space, and enzymatic degradation in microglia or astrocytes, although the details are still unknown [4,5]. Recently, “the glymphatic system” was proposed as a lymphatic system in the brain that may excrete large molecules in the brain parenchyma into the cerebral spinal fluid [6]. In support of this model, Illif et al. reported that Aβ_1–40_, which was labeled fluorescently or with radioisotopes and was injected into the striatum, was observed around capillaries and drainage veins [6]. The clearance of Aβ was decreased in AQP4 null mice, suggesting that perivascular water-flow is involved. However, the physiological roles and detailed mechanisms of this system still hypothetically lack dynamic data on in vivo flow [7,8].

In contrast, deposits of Aβ in the penetrating arteries and leptomeningeal arteries are pathologically found in cases with cerebral amyloid angiopathy (CAA) [9]. CAA is an age-related disease most often found with AD [10]. The Aβ peptide is known to be generated by sequential cleavage of the amyloid precursor protein in neurons in the cerebral cortex and may be transported to the penetrating arteries and leptomeningeal arteries [10]. Unlike the senile plaques of AD, the deposits are dominated by the shorter peptide fragments, suggesting the difference in deposition rate and transportation among peptides [11].

In the present study, we evaluated the dynamics of Aβ oligomers or fibrils in vivo in mice using two-photon microscopy.

## 2. Materials and Methods

### 2.1. Animal Preparation

All experiments described in this study were approved by the Osaka City University Ethics Committee on Animal Resources (Protocol #21030). The animal experimentation was conducted following the protocol and ARRIVE criteria.

Tie2 is a receptor of angiopoietins 1 and 2 and is expressed specifically in all endothelial cells throughout development and in adults [12,13]. Female and male mice at the ages of 8 and 12 weeks expressing green fluorescent protein (GFP) at the vascular endothelial cell Tie2 (STOCK Tg [Tie2-GFP] 287 Sato/J, the Jackson Laboratory, Bar Harbor, ME, USA) were used to detect Aβ in the perivascular space. Unless otherwise noted, C57BL/6 mice (CLEA Japan, Inc., Tokyo, Japan) were used. Mice were maintained on a 12 h light/dark cycle with humidity and temperature controlled at normal levels and allowed food (CLEA Rodent Diet CE-2) and water ad libitum.

In all experiments, animals were kept anesthetized with 1.5 to 2.0% isoflurane inhalation. After incising the scalp and exposing the skull, a 4 mm diameter cranial window was installed using a dental drill. A solution of Aβ (oligomers or fibrils), dextran, or both was applied topically on the brain surface. The cranial window was closed with a cover glass, and repeated imaging began 30 min after the application of the solution.

In all experiments described below, animals were excluded from the experiments only when the preparation was not good enough for the observation. For experiments requiring statistical analysis, at least 5 animals were employed (detailed number for each experiment was described in the Section 2).

### 2.2. Aβ and Dextran Solutions

HiLyte Fluor 647-labeled Aβ_1–40_ was obtained from AnaSpec (San Jose, CA, USA) and kept frozen at −20 °C. Aβ solution was prepared on ice, first with 0.1% NH_4_OH to 1 mM and then with phosphate-buffered solution (PBS) to a final concentration of 100 μM. After preparation, the solution was aliquoted in 10 µL portions on ice and immediately frozen at −80 °C as a sample for the Aβ oligomer. The Aβ fibril samples were prepared by shaking the Aβ solution at 37 °C for 6 days at 1000 rpm. TRITC-dextran (40 kD), FITC-dextran (40 kD) (TdB Labs, Ultuna, Sweden), and TRITC-dextran (4.4 kD) (Sigma–Aldrich, St. Louis, MO, USA) were dissolved in PBS to 10–100 μM.

Fibril formation of Aβ_1–40_ was confirmed with transmission electron microscopy (TEM). The solution was dropped on Formvar/Cu grids with mesh 200 (VECO GRID H200, VECO, Eerbeek, The Netherlands). After 3 min, the grids were cleaned in water for 60 s and then negatively stained with 1% (*w*/*v*) uranyl acetate for 60 s. Images were taken with TEM (Talos F200CG2, ThermoFisher Scientific, Waltham, MA, USA) at an acceleration voltage of 80 kV.

### 2.3. In Vivo Observation with Two-Photon Microscopy

The dynamic movement of Aβ oligomers/fibrils was observed with a two-photon laser microscope (A1RMP+1080, Nikon, Tokyo, Japan) equipped with a pulse laser, Chameleon Vision II (Coherent, Santa Clara, CA, USA), of which the pulse width was 140 fs and the repetition rate was 80 MHz. Mice were immobilized on the stage below the microscope under isoflurane anesthesia. Fluorescent images were obtained with green (center wavelength 525 nm, bandwidth 50 nm) and pink (center wavelength 629 nm, bandwidth 56 nm) bandpass filters with an excitation wavelength of 920 nm.

Dynamic three-dimensional images were taken at 30 min intervals from 30 min to 180 min after the amyloid solution was placed. Three-dimensional images were reconstructed with NIS-Elements software (Nikon, Tokyo, Japan).

Capillary accumulation of Aβ oligomers/fibrils was measured at depths of 50, 100, and 150 μm from the cortical surface. The number of Aβ-positive capillaries was counted in a 509 μm square and statistically analyzed following the methods described below (*n* = 5 for each condition). To elucidate specific Aβ dynamics in the cerebral parenchyma, the accumulation of dextran of different sizes (4.4 kD and 40 kD) was also measured.

### 2.4. Statistical Analysis

The number of Aβ-positive capillaries was counted at depths of 50, 100, and 150 μm at 30 min intervals from 30 min to 180 min (*n* = 5 for each condition). Normality of the data obtained for each condition was evaluated with a Shapiro–Wilk test using IBM-SPSS (Tokyo, Japan). After confirming normal distribution in all datasets, two-way repeated measures analysis of variance was used to evaluate time-dependent accumulation of Aβ together with effect of observation depths.

## 3. Results

### 3.1. Fibril Formation of Aβ_1–40_

**Figure 1:**
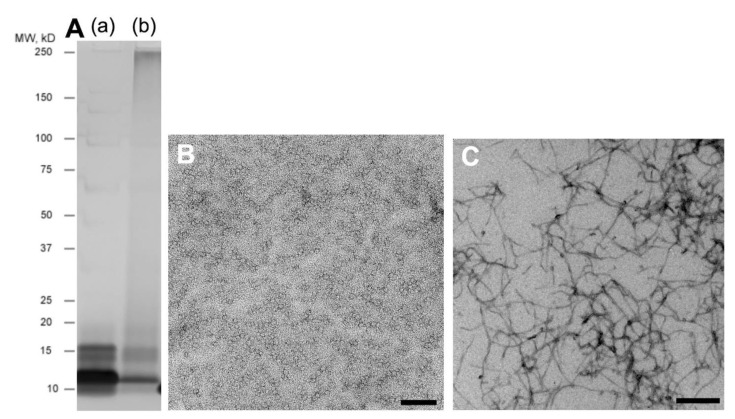
Fibril formation of Aβ_1–40_. (**A**). SDS–PAGE of Aβ_1–40_ solution immediately after being prepared with silver staining on ice (**a**) or solution after incubation for 144 h (**b**). Fresh sample (**a**) showed strong bands at MW 10–16 kD, suggesting a low number of oligomers, especially monomers, dimers, and trimers, whereas incubated sample (**b**) showed a strong band at MW 250 kD or higher. TEM of the fresh solution showed granular structures of 9.0–13.8 nm in diameter (**B**), whereas that of the incubated solution showed a fibrous structure of approximately 25 nm in diameter (**C**). Scale bar: (**B**) 100 nm; (**C**) 500 nm.

SDS–PAGE of Aβ_1–40_ solution immediately after being prepared with silver staining on ice showed strong bands at MW 10–16 kD, suggesting a low number of oligomers, especially monomers, dimers, and trimers (Figure 1A(a)). TEM of the same solution showed granular structures of 9.0–13.8 nm in diameter (Figure 1B). In contrast, SDS–PAGE of the solution after incubation for 144 h showed a strong band at MW 250 kD or higher (Figure 1A(b)). TEM of the solution showed a fibrous structure approximately 25 nm in diameter (Figure 1C).

### 3.2. Perivascular Distribution of Aβ_1–40_

**Figure 2:**
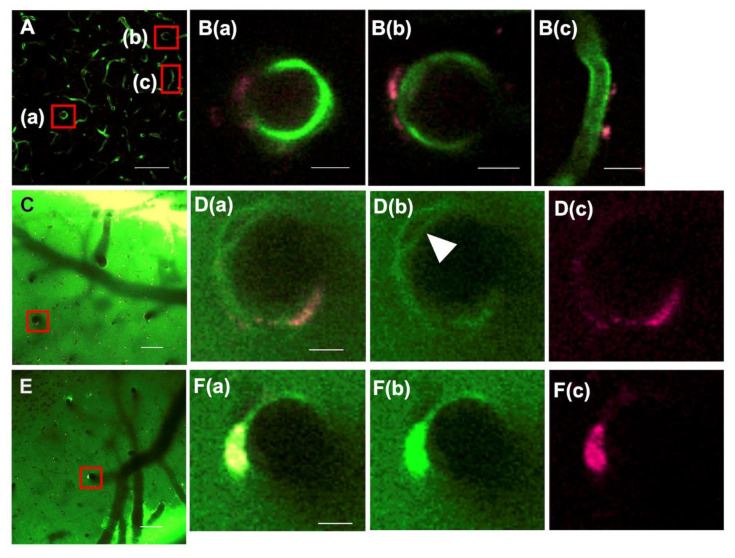
Distribution of tracers transported from the cortical surface. (**A**,**B**): The Hilyte Fluor 647-labeled Aβ_1–40_ oligomer was found around a penetrating artery (**A**(**a**)), a penetrating vein (**A**(**b**)), and a capillary (**A**(**c**)) in a Tie2-GFP mouse. (**B**(**a**–**c**)) are enlarged images of red squares (**A**(**a**–**c**)), respectively. Notably, Aβ accumulated at only part of the vessel wall, not at the entire circumference, in all vessels observed. (**D**) enlarged from a red square in (**C**): FITC-dextran of 40 kD distributed over the entire circumference of a penetrating artery as well as in the parenchyma (**D**(**b**)). A double barrel was also observed (arrowhead). In contrast, the accumulation of Aβ oligomer was localized to part of the circumference (**D**(**c**)), which is evident in a merged image (**D**(**a**)). (**F**) enlarged from a red square in (**E**): Massive accumulation of Aβ fibrils was noticed at a penetrating vein (**F**(**c**)), whereas 40 kD dextran was distributed more widely (**F**(**a**,**b**)), a merged image of (**F**(**b**,**c**)). Scale bar: (**A**,**C**,**E**) 100 μm; (**B**,**D**,**F**) 10 μm.

Aβ_1–40_ oligomer solution labeled with HiLyte Fluor 647 was placed on the cortical surface (*n* = 5). After 60 min, Aβ was found around penetrating arteries and veins in a Tie2-GFP mouse (Figure 2A,B). Most notably, Aβ accumulated at only part of the vessel wall, not at the entire circumference, in all vessels observed. Capillary accumulation of Aβ was also noticed, suggesting parenchymal transportation of Aβ, as there is no perivascular space around the capillary.

Coadministration of Aβ_1-40_ oligomers and 40 kD dextran (*n* = 6) showed that Aβ accumulation was localized to part of the circumferential perivascular space, whereas dextran was distributed over the entire circumference, including the double barrel structure, as well as in the cortical parenchyma (Figure 2C,D). Compared to Aβ_1–40_ oligomers, Aβ fibrils accumulated more locally in a large mass (*n* = 12) (Figure 2E,F).

### 3.3. Dynamic Perivascular Transportation of Aβ

**Figure 3:**
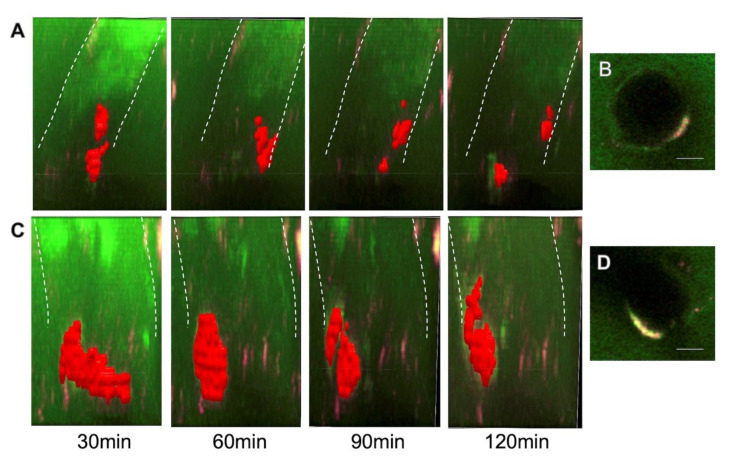
Dynamic perivascular transportation of HiLyte Fluor 647-labeled Aβ_1–40_ oligomers (**A**,**B**) and fibrils (**C**,**D**). The perivascular distribution of Aβ was captured in horizontal planes (**B**,**D**) and reconstructed in 3 dimensions every 30 min (**A**,**C**). FITC-labeled dextran at 40 kD was distributed in the entire circumference of penetrating vessels, whereas Aβ accumulated in only localized perivascular spaces (**B**,**D**). The accumulated mass of Aβ oligomers moved around the vessel wall and separated (**A**). Aβ fibrils formed a large mass and elongated in the direction of a penetrating vessel (**C**). Scale bar: (**B**,**D**) 10 μm. Three-dimensional images and dynamic motion are shown in Supplementary Videos.

As shown in Figure 3A,C, accumulated Aβ oligomers/fibrils moved along the penetrating vessels in the perivascular space. The speed of its transportation was mostly very slow, less than 10 μm/min. In addition, accumulated Aβ changed its shape and size, suggesting assembly and disassembly of Aβ molecules to the mass over time. Separation and fusion of the accumulated Aβ mass may also suggest loose binding of the masses. In addition, transportation along the penetrating vessels was slower, and the transformation of its shape was less prominent in fibrils than in oligomers (Figure 3A,C).

### 3.4. Capillary Accumulation of Aβ in the Parenchyma

**Figure 4:**
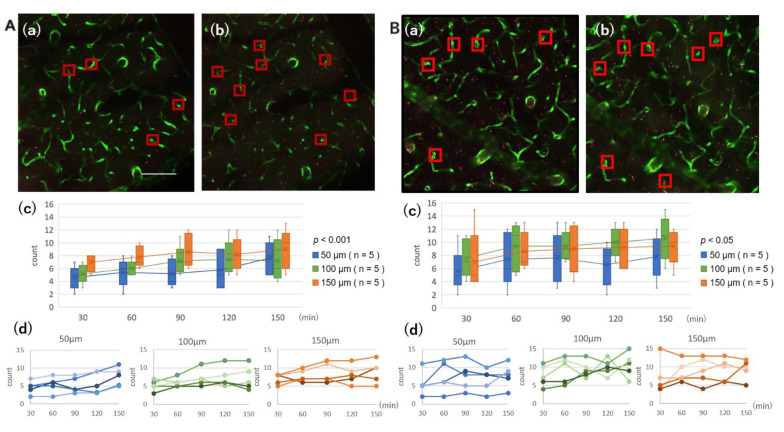
Both Aβ oligomers (**A**) and fibrils (**B**) accumulated progressively in parenchymal capillaries. The number of capillaries with Aβ accumulation, indicated with squares in Panels (**a**,**b**), in a 509 μm square was counted at depths of 50, 100, and 150 μm every 30 min from 30 min (**a**) to 150 min (**b**). In contrast with the planar distribution of Aβ in the penetrating vessels, Aβ accumulated in a spot along the capillaries. Progressive accumulation was significant both in the oligomers (*p* < 0.001) and in the fibrils (*p* < 0.05) (**A**(**c**,**d**),**B**(**c**,**d**)). (**A**(**c**),**B**(**c**)) are box-and-whisker plots of data shown in (**A**(**d**),**B**(**d**)), respectively. The speed of accumulation was faster in oligomers than in fibrils. The Aβ accumulation measured at 3 depths was not statistically different between them. Scale bar: (**A**) 100 μm.

As shown in Figure 4, both Aβ oligomers and fibrils accumulated progressively in parenchymal capillaries. In contrast with the planar distribution of Aβ in the penetrating vessels, Aβ accumulated in a spot along the capillaries. Although progressive accumulation was significant both in the oligomers (*p* < 0.001) as well as in the fibrils (*p* < 0.05), the speed of accumulation was generally faster in oligomers than in fibrils (Figure 4). Effect of observation depths in the two-way ANOVA was not statistically significant, suggesting that the Aβ accumulation measured at three depths was not different between them.

### 3.5. Perivascular Flow of Large Dextran

**Figure 5:**
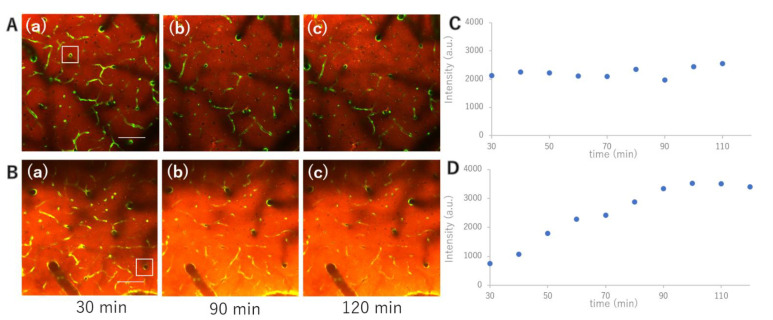
Dynamic transportation of TRITC-dextran at 4.4 kD (**A**) and 40 kD (**B**) was measured in a Tie2-GFP mouse. Progressive accumulation of 40 kD dextran in the perivascular space was observed (**B**), whereas 4.4 kD dextran reached a plateau within 30 min after topical application (**A**) ((**a**) 30 min, (**b**) 90 min, (**c**) 120 min after the dextran administration). Fluorescent intensity of squares in (**A**(**a**)),(**B**(**a**)) was periodically measured ((**C**,**D**), respectively). Scale bar: (**A**(**a**),**B**(**a**)) 100 μm.

The dynamic transportation of TRITC-dextran at 4.4 kD (*n* = 6) and 40 kD (*n* = 6) was measured in a Tie2-GFP mouse (Figure 5). Progressive accumulation of 40 kD dextran in the perivascular space was observed (B), whereas 4.4 kD dextran reached a plateau within 30 min following topical application (A).

## 4. Discussion

In the present study, we demonstrated that Aβ placed on the cortical surface was slowly transported to the deeper parenchyma through the perivascular space. These results suggest that Aβ peptide, generated by sequential cleavage of the amyloid precursor protein in neurons in the cerebral cortex, can be transported to the penetrating arteries and leptomeningeal arteries. Facilitation of Aβ excretion through the system may prevent oligomerization of Aβ or formation of senile plaque in the cortex, preventing the development of AD. Until now, inhibition of Aβ production by γ-secretase inhibitors was unsuccessful [14], whereas therapies utilizing antibodies to Aβ have been only partially successful [15]. As oligomerization of Aβ is now regarded as the first step in AD development, enhanced wash-out of Aβ from the cerebral cortex through the perivascular space might reduce toxicity of the Aβ [16].

Compared to 40 kD dextran, which was distributed in the entire circumference of the vessels, Aβ was found to be localized to part of the perivascular space. Previous reports suggested that Aβ may be transported via the perivascular space without clear images demonstrating a detailed distribution [6,17]. The present study is the first to show with live imaging that Aβ may locally accumulate in the perivascular space of both the penetrating artery and vein.

We also demonstrated that the accumulated Aβ mass moves along the penetrating vessels, changing its shape with separation and fusion. The speed of Aβ mass transportation is very slow and can sometimes even move backward. Aβ is known to polymerize easily with alterations in rheological characteristics [18]. The assembly and disassembly of Aβ molecules may be involved in the change in Aβ mass form.

In the present study, the direction of Aβ transportation was not steady, with both penetrating arteries and veins involving Aβ accumulation from the surface. The direction of perivascular flow is controversial; some suggest arterial influx and venous efflux [6] and others suggest efflux in both arteries and veins [19].

The difference in the direction and speed of perivascular flow and in the detailed structural pathway may depend on the molecules used in the experiments, i.e., dextran [6], ovalbumin [6], and QDot655 [20]. Their molecular weight is 0.58 to 45 kD without the capability of polymerization. In the present study, dextran at 4.4 kD and 40 kD was compared to Aβ, showing that small tracers can diffuse through the parenchyma. At the same time, large molecules may be transported from the subarachnoid space through the circumferential perivascular space along penetrating vessels to the parenchyma. Polymerization with high shear stress may involve the difference in transportation and distribution of Aβ and other tracers. The larger mass and slower transportation in Aβ fibrils than in Aβ oligomers in the present study may support this hypothesis.

We also demonstrated that Aβ increasingly accumulated around the capillary in the cortical parenchyma. Since capillaries have no perivascular space, the result may indicate that Aβ may be transported from the perivascular space to the parenchyma, indicating that the reversed flow may be involved in the physiological clearance of Aβ.

The limitations of the present study include the following: (1) general anesthesia with isoflurane, which is known to increase intracranial pressure and suppress perivascular transportation [21], was used, (2) transportation of Aβ from the parenchyma to the cortical surface was not assessed, (3) the initial period immediately after Aβ application was not evaluated, (4) labeling with fluorescent dye may affect the physical character of Aβ and (5) Aβ_1-42_ compared to Aβ_1–40_ may behave differently. Although none of these limitations affected the significance of the present study, further studies are warranted.

In conclusion, the present study demonstrated that Aβ placed on the cortical surface was slowly transported to the deeper parenchyma through the perivascular space. The first-ever visualization of Aβ transportation indicates that loose polymerization may affect its transportation.

## Supplementary Materials

The following supporting information can be downloaded at: https://www.mdpi.com/article/10.3390/ijms23126422/s1.
