# Supplementary material for: In Vivo Dynamic Movement of Polymerized Amyloid β in the Perivascular Space of the Cerebral Cortex in Mice
Source: Int J Mol Sci. 2022 Jun 8;23(12):6422. doi: 10.3390/ijms23126422 (PMC9223597; doi:10.3390/ijms23126422)
Supplement: Supplementary file 1 [file ijms-23-06422-s001.zip › Legends to Supplementary Movie.pdf]

Legends to Supplementary Movie

Supplementary Movie S1. A reconstructed 3-dimensional video showing accumulated amyloid $\beta_{1\text{-}40}$ oligomers in the perivascular space of a penetrating vessel. The accumulated mass seems thin, 1 μm thick, in the lateral view and flat, 30 μm long and 20μm wide, in the front view. The image was captured at 60 minutes after the amyloid application (Figure 3A, B).

Supplementary Movie S2. A reconstructed 3-dimensional video showing accumulated amyloid β1-40 (Aβ) fibrils in the perivascular space of a penetrating vessel. The accumulated mass of Aβ fibrils was thicker in the lateral view and larger in the front view than that of Aβ oligomers. The image was captured at 30 minutes after the amyloid application (Figure 3C, D).

Supplementary Movie S3. A reconstructed time-lapse image of amyloid $\beta_{1\text{-}40}$ (Aβ) oligomers in the perivascular space of a penetrating vessel. Separated Aβ masses convened at 60 minutes and became separated again after 90 minutes. Scale bar 10 μm.

Supplementary Movie S4. A reconstructed time-lapse image of amyloid $\beta_{1\text{-}40}$ (Aβ) fibrils in the perivascular space of a penetrating vessel. A large Aβ mass elongated in the direction of the vessel at 60 minutes and became separated after 90 minutes. Scale bar 10 μm.
